# Crystallographic Insight into Collagen Recognition by Discoidin Domain Receptor 2

**DOI:** 10.1016/j.str.2009.10.012

**Published:** 2009-12-09

**Authors:** Federico Carafoli, Dominique Bihan, Stavros Stathopoulos, Antonios D. Konitsiotis, Marc Kvansakul, Richard W. Farndale, Birgit Leitinger, Erhard Hohenester

**Affiliations:** 1 Department of Life Sciences, Imperial College London, London SW7 2AZ, UK; 2 Department of Biochemistry, University of Cambridge, Cambridge CB2 1QW, UK; 3 Division of National Heart and Lung Institute, Imperial College London, London SW7 2AZ, UK

**Keywords:** PROTEINS, CELLBIO

## Abstract

The discoidin domain receptors, DDR1 and DDR2, are widely expressed receptor tyrosine kinases that are activated by triple-helical collagen. They control important aspects of cell behavior and are dysregulated in several human diseases. The major DDR2-binding site in collagens I–III is a GVMGFO motif (O is hydroxyproline) that also binds the matricellular protein SPARC. We have determined the crystal structure of the discoidin domain of human DDR2 bound to a triple-helical collagen peptide. The GVMGFO motifs of two collagen chains are recognized by an amphiphilic pocket delimited by a functionally critical tryptophan residue and a buried salt bridge. Collagen binding results in structural changes of DDR2 surface loops that may be linked to the process of receptor activation. A comparison of the GVMGFO-binding sites of DDR2 and SPARC reveals a striking case of convergent evolution in collagen recognition.

## Introduction

Supramolecular collagen assemblies are crucial for the mechanical stability of animal bodies (Myllyharju and Kivirikko, 2004). The basic collagen structure is a triple helix of three chains containing multiple Gly-X-X' repeats; X and X' are often proline and 4-hydroxyproline (Hyp, O), respectively (Brodsky and Persikov, 2005). Apart from their prominent structural roles, collagens have fundamental functions in cell adhesion and signaling, by serving as ligands for a diverse set of cellular receptors (Heino, 2007; Leitinger and Hohenester, 2007). The most widely distributed collagen receptors are a subclass of β1 integrins and two homologous receptor tyrosine kinases (RTKs), the discoidin domain receptors, DDR1 and DDR2. While collagen binding and signaling by integrins are understood in atomic detail (Emsley et al., 2000; Hynes, 2002), much less is known about the DDRs. Binding of triple-helical collagen to DDRs results in slow and sustained receptor phosphorylation (Shrivastava et al., 1997; Vogel et al., 1997), ultimately regulating many aspects of cell proliferation, adhesion, and migration, as well as remodeling of the extracellular matrix (Vogel et al., 2006). Mice lacking DDR1 exhibit defective mammary gland development (Vogel et al., 2001), kidney function (Gross et al., 2004), and arterial wound repair (Hou et al., 2001). Mice lacking DDR2 exhibit dwarfism resulting from reduced chondrocyte proliferation (Kano et al., 2008; Labrador et al., 2001); a similar phenotype is observed in human patients with mutations in the *DDR2* gene (Bargal et al., 2009). Aberrant DDR function in humans is also associated with osteoarthritis, fibrosis, and cancer (Vogel et al., 2006).

Structurally, the DDRs are characterized by an extracellular region consisting of a discoidin (DS) domain that is followed by a domain unique to DDRs, a transmembrane helix, a large cytoplamic juxtamembrane region, and, finally, a C-terminal kinase domain. Several loops within the DS domain have been shown to be essential for collagen binding (Abdulhussein et al., 2004; Ichikawa et al., 2007; Leitinger, 2003), but how collagen is recognized has remained unknown. We recently identified a GVMGFO motif as the major DDR2-binding site in collagens I–III (Konitsiotis et al., 2008). Here, we report the crystal structure of the DS domain of human DDR2 bound to a triple-helical collagen peptide containing this motif. The structure reveals that the apolar GVMGFO motifs of two collagen chains are recognized by an amphiphilic pocket in DDR2, in a manner that is fundamentally different from the metal ion-dependent mechanism employed by integrins.

## Results

### Crystal Structure of a DDR2 DS Domain-Collagen Peptide Complex

**Figure 1:**
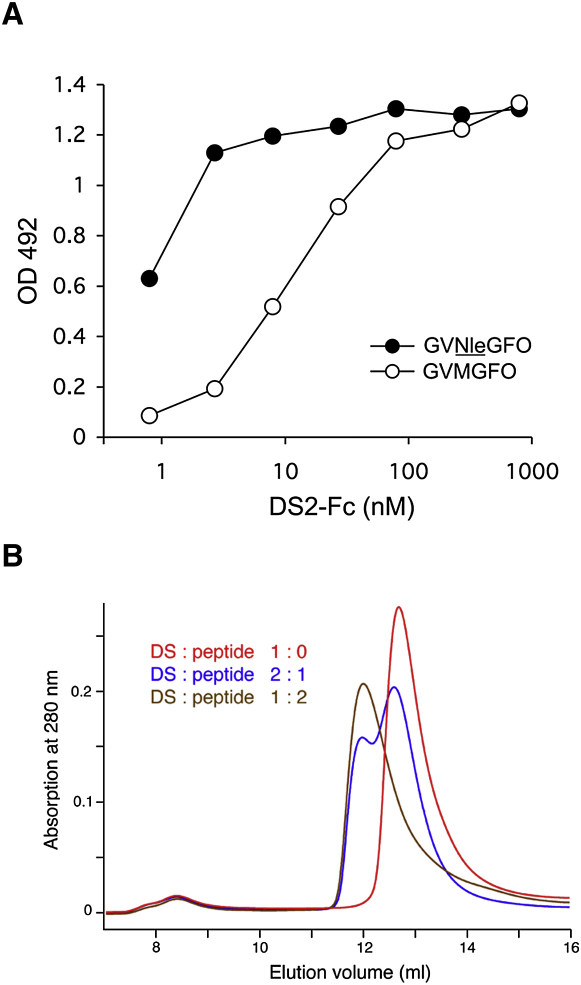
Collagen Peptide Binding by the DDR2 DS Domain (A) Solid-phase binding assay with recombinant DS2-Fc protein (Leitinger, 2003) added to 96-well plates coated with triple-helical collagen peptides at 10 μg/ml: GPC-(GPP)_5_-GPRGQOGVXGFO-(GPP)_5_-GPC-NH_2_, where X is either methionine or norleucine. Shown is a representative of three independent experiments, each performed in duplicate. (B) Analytical size exclusion chromatograms of the free DDR2 DS domain and its complex with the triple-helical collagen peptide Ac-GPOGPOGPOGPR-GQOGVNleGFOGPOGPOG-NH_2_. The DS domain and peptide were mixed in the indicated molar ratios. A globular molecular mass standard of 29 kDa, carbonic anhydrase, elutes at 12.3 ml from this column.

**Figure 2:**
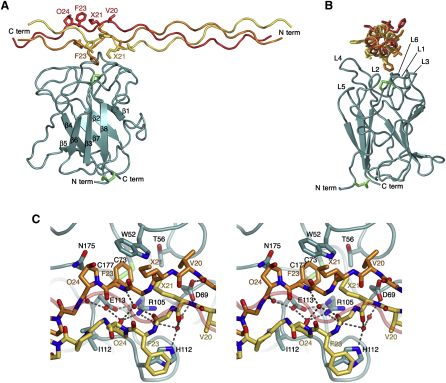
Crystal Structure of the DDR2 DS Domain-Collagen Complex (A) Cartoon representation of the DS domain (cyan) and the collagen peptide (yellow, leading chain; orange, middle chain; red, trailing chain). The β strands of the DS domain are numbered sequentially. Disulfide bonds are in green. The side chains of the collagen GVMGFO motif are shown as sticks. Selected residues are labeled. X denotes norleucine. (B) Orthogonal view of the complex, related to (A) by a 90° rotation about a vertical axis. The collagen peptide is viewed from N to C terminus. Loops at the top of the DS domain are labeled as follows: L1-3, β1-β2; L4, β3-β4; L5, β5-β6; and L6, β7-β8. (C) Stereo view of the DDR2-collagen interface. Selected DDR2 and collagen residues are shown as sticks, in the same colors as in (A). The trailing collagen chain is shown as a semitransparent coil. Water molecules are shown as red spheres. Dashed lines indicate hydrogen bonds.

During the course of our previous study (Konitsiotis et al., 2008), we discovered that substitution of methionine in GVMGFO by the isosteric amino acid norleucine (Nle) increases DDR2 binding in a solid-phase assay ∼10-fold (Figure 1A). We synthesized a number of short triple-helical peptides for co-crystallization with the DDR2 DS domain. The peptides contained the DDR2-binding sequence, GPRGQOGVNleGFO, flanked by 2–3 GPO repeats at either end; the GPRGQO sequence was included because it is required for DDR2 activation in cells (Konitsiotis et al., 2008). Since we obtained crystals with the first peptide tested, Ac-GPOGPOGPOGPRGQOGVNleGFOGPOGPOG-NH_2_, we did not perform a systematic analysis of the remaining peptides. We used analytical size exclusion chromatography to demonstrate peptide binding to the DDR2 DS domain in solution (Figure 1B). The free DS domain (molecular mass, 20.1 kDa) eluted as a single peak at 12.7 ml, corresponding to a monomer. When the triple-helical collagen peptide Ac-GPOGPOGPOGPRGQOGVNleGFOGPOGPOG-NH_2_ (molecular mass, 7.9 kDa) was added in a two-fold molar excess, a protein-peptide complex was formed that eluted at 12.0 ml (note that the peptide does not contribute to the absorption at 280 nm). This elution volume is consistent with a complex of 1:1 stoichiometry. Unlike the free DS domain, which is not very soluble, the DS-collagen peptide complex could be concentrated to 10 mg/ml and crystallized. Diffraction data to 1.6 Å resolution were collected using synchrotron radiation and the structure of the DDR2 DS-collagen peptide complex was solved by molecular replacement (Figure 2; Table 1).

The DDR2 DS domain is an eight-stranded β-barrel arranged in two antiparallel β sheets, as described previously (Ichikawa et al., 2007). The N and C termini are located at the flat bottom of the barrel, connected by the disulfide bridge between Cys30 and Cys185. At the top of the barrel, five protruding loops (L1–L3 connecting β1 to β2, L4 connecting β3 to β4, and L6 connecting β7 to β8) create a trench that accommodates the collagen peptide; the disulfide bridge between Cys73 and Cys177 lies at the bottom of this trench. The location of the collagen binding site at the top of the DDR2 DS domain is in agreement with results from previous mapping studies (Ichikawa et al., 2007; Leitinger, 2003).

The DDR2 DS domain interacts with two of the three GVNleGFO motifs in the C-terminal part of the 80 Å–long collagen peptide, burying 530 Å^2^ of solvent-accessible collagen surface with good shape complementarity (surface complementarity score, 0.71; program SC) (CCP4, 1994). The three chains of a collagen triple helix are arranged with a characteristic one-residue stagger (Brodsky and Persikov, 2005). In the DDR2 DS-collagen structure, the leading and middle chains of the collagen peptide (for a definition, see Emsley et al., 2000) account for 40% and 60% of the interface, respectively; the trailing chain is not involved in DDR2 binding.

All the major DDR2-peptide contacts are made with the GVNleGFO motifs of the leading and middle chains, but the following GPO triplet of the middle chain (GPK in native collagens I–III) is also within van der Waals distance of DDR2. The central feature of the DDR2-collagen interface is an amphiphilic pocket at the top of the DS domain that accommodates Nle21 (leading chain) and Phe23 (middle chain) (Figure 2C; for consistency we use the same numbering scheme for the collagen peptide as in our previous work on SPARC; Hohenester et al., 2008). One wall and the floor of the pocket are essentially apolar (Trp52, Thr56, and Cys73-Cys177 disulfide bridge), whereas the other wall is dominated by three charged residues (Asp69, Arg105, and Glu113) interacting with a string of water molecules. A salt bridge between Arg105 and Glu113 forms two hydrogen bonds with the hydroxyl group of Hyp24 (leading chain) and contacts one edge of the phenyl ring of Phe23 (middle chain), suggestive of a C-H⋯π hydrogen bond (Brandl et al., 2001). The pocket is completed by Asp69, which hydrogen bonds with the collagen backbone, and Asn175. At one rim of the pocket, His110 and Ile112 interact with Phe23 and Hyp24 of the leading chain, respectively.

**Figure 3:**
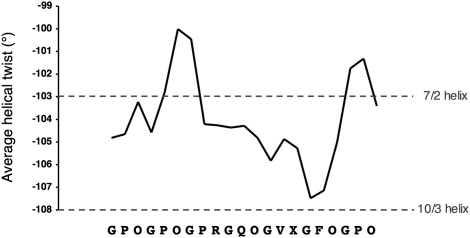
Helix Parameters of the Collagen Peptide Residues [*i*–1 (leading), *i* (middle), *i*+1 (trailing)] were fitted to residues [*i* (leading), *i*+1 (middle), *i*+2 (trailing)], and the associated rotation was taken as the helical twist at position *i*. The sequence of the collagen peptide is indicated at the bottom. X denotes norleucine. The twists of ideal left-handed 7/2 and 10/3 helices are −103° and −108°, respectively (Okuyama et al., 2006).

The collagen peptide in the DDR2 DS-collagen complex is completely straight (Figure 2B), and the helical parameters of the three collagen chains are therefore essentially identical. The GPO-rich N-terminal region is close to a 7/2 helical symmetry, whereas the GVNleGFO motif approximates a more relaxed 10/3 symmetry (Figure 3). A relaxation of the helical twist in regions lacking imino acids has been observed in several model peptide structures (Kramer et al., 1999; Okuyama et al., 2006). The observed transition in helix parameters is thus likely to be an inherent feature of the collagen peptide itself, rather than a consequence of DDR2 binding.

The details of collagen binding revealed by our structure are in excellent agreement with biochemical results showing that M, F, and O of the GVMGFO motif are critical for DDR2 binding (Konitsiotis et al., 2008). Why the substitution of methionine by norleucine enhances DDR2 binding is not evident from the structure, as the slightly longer methionine side chain is readily modeled into the amphiphilic pocket without steric clashes (not shown). It may be significant, however, that there is a close (3.5 Å) contact between Cδ of Nle21 (leading chain) and Phe23 (middle chain). This contact may be less favorable when the corresponding atom is sulfur, as it is in methionine. In any case, the effect is subtle, and we think that it is highly unlikely that methionine is recognized in a radically different manner. We will therefore make no distinction between norleucine and methionine in the following discussion.

An arginine four residues upstream of the GVMGFO motif has been shown to contribute to DDR2 binding, and the GPR triplet containing this arginine is strictly required for signaling (Konitsiotis et al., 2008). In our structure, the side chain of Arg15 (trailing chain) points toward an acidic patch on the DS domain formed by Glu66, Glu67, and Asp69, but it is too distant (8 Å) to form any specific interactions (not shown). This long-range electrostatic interaction may explain why Arg15 contributes to DDR2 binding. However, further studies are required to understand the apparently critical role of this arginine in receptor activation (Konitsiotis et al., 2008).

### Comparison with the Solution Structure of the Free DDR2 DS Domain

**Figure 4:**
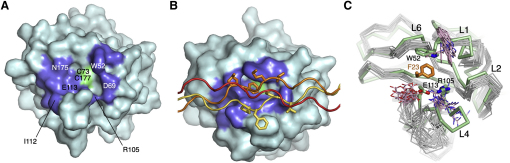
Structural Changes in the DDR2 DS Domain upon Collagen Binding (A) Surface representation of the free DDR2 DS domain in solution (Ichikawa et al., 2007). The collagen-binding residues identified by this study are in green (disulfide) and blue (all other residues). Selected residues are labeled. (B) Surface representation of the DS domain in complex with the collagen peptide (yellow, leading chain; orange, middle chain; red, trailing chain). The side chains of the GVMGFO motif are shown as sticks. The view direction and coloring of the DS domain surface are the same as in (A). (C) Superposition of the NMR ensemble of the free DDR2 DS domain (Ichikawa et al., 2007) (gray Cα traces; Trp52, Arg105, and Glu113 side chains in pink) and the crystal structure of the DDR2 DS-collagen (green, DS domain; orange, Phe23 of the collagen middle chain). The structures were superimposed using 43 Cα atoms of the DS domain β-barrel (rmsd 0.82 Å). Model 5 of the NMR ensemble is most similar overall to the crystal structure (rmsd 1.8 Å) and was taken as the reference. Selected residues and loops are labeled.

The solution structure of the free DDR2 DS domain has been determined, and the collagen-binding site has been identified by transferred cross-saturation experiments and mutagenesis (Ichikawa et al., 2007). Although there is good general agreement with our DS-collagen complex structure regarding the identity of the major collagen-binding residues (Figures 4A and 4B), the mode of collagen binding was not predicted correctly. The presence of Arg105 and Glu113 in the collagen-binding site of DDR2 led Ichikawa et al. (2007) to predict that complementary charges must exist in collagen. However, the GVMGFO motif is notably apolar, and charge compensation in the DDR2-collagen complex is, in fact, achieved by a buried salt bridge between Arg105 and Glu113 (Figure 2C). A comparison of our complex structure with the NMR ensemble of the free DS domain shows that collagen binding leads to a restructuring of loops L1 (bearing the critical Trp52) and L4 (bearing at its base Arg105 and Glu113). L1 and L4 appear to move in a concerted manner to clamp down on Phe23 of the collagen middle chain (Figure 4C). The movement of L4 is followed by L5 at the side of the DS domain β-barrel (not shown). A caveat of this comparison is that there are very few long range NOEs that determine the conformations of L1 and L4 in the NMR ensemble. Nevertheless, it is likely that collagen binding leads to a freezing of the mobile loops surrounding the collagen-binding trench.

### Conservation of Collagen-Binding Residues in DDR1 and DDR2

**Figure 5:**
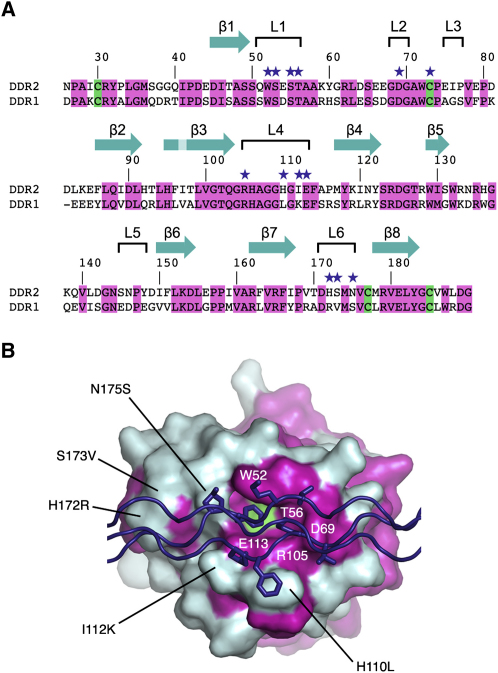
Sequence Conservation of the Collagen-Binding Site (A) Sequence alignment of the DS domains of human DDR1 and DDR2. The sequence numbering and secondary structure elements of the DDR2 DS domain are indicated above the alignment. Conserved residues and cysteines are highlighted in magenta and green, respectively. Residues that lose ≥5 Å^2^ of their solvent-accessible surface upon collagen binding are indicated by purple stars. (B) Mapping of conserved residues onto the molecular surface of the DDR2 DS domain. Residues that are identical in DDR1 and DDR2 are in magenta. The Cys73-Cys177 disulfide bridge is in green. Selected conserved residues and nonconserved substitutions in DDR1 are indicated. The collagen peptide is in purple, and the side chains of the GVMGFO motifs of the leading and middle chains are shown as sticks.

The central collagen-binding residues of DDR2 delineated by our structure (Trp52, Thr56, Asp69, Arg105, Glu113, and Cys73-Cys177 disulfide bridge) are strictly conserved in DDR1, consistent with the binding of both receptors to fibrillar collagens (Shrivastava et al., 1997; Vogel et al., 1997) and to GVMGFO-containing peptides (authors' unpublished data). Notably, however, several collagen-binding residues outside of the amphiphilic pocket are not conserved: DDR2 residues His110, Ile112, and Asn175 are replaced in DDR1 by Leu110, Lys112, and Ser175, respectively (Figure 5). These substitutions may be responsible for the distinct specificities of the two homologous receptors, such as the exclusive binding of DDR1 to basement membrane collagen IV (Shrivastava et al., 1997; Vogel et al., 1997) and of DDR2 to collagen X (Leitinger and Kwan, 2006).

**Figure 6:**
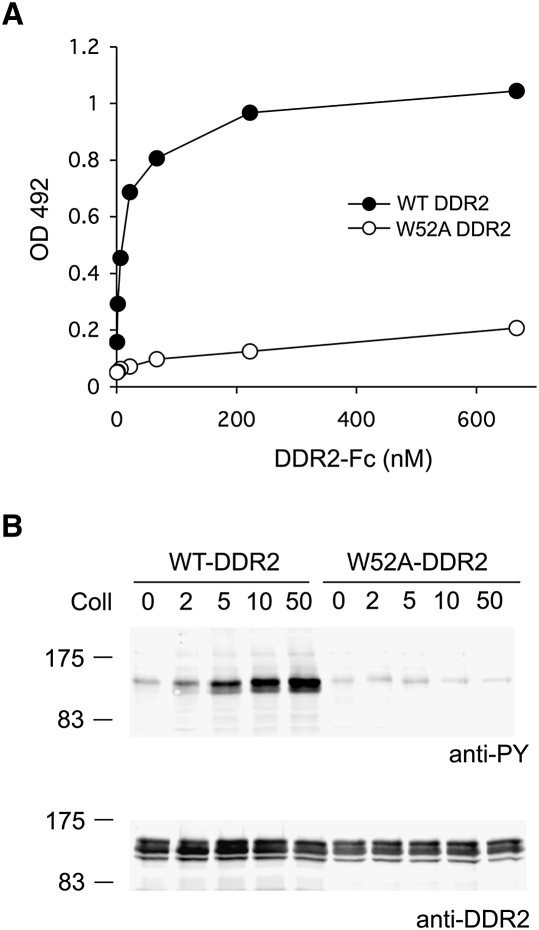
Essential Role of Trp52 in DDR2 Function (A) Solid-phase binding assay with recombinant wild-type or W52A DDR2-Fc proteins added for 3 hr at room temperature to 96-well plates coated with either collagen I or BSA. Shown is a representative of three independent experiments, each performed in duplicate. (B) Full-length wild-type or W52A DDR2 was transiently expressed in HEK293 cells. After stimulation for 90 min with collagen I (Coll), aliquots of cell lysates were analyzed by SDS-PAGE and Western blotting. The blots were probed with anti-phosphotyrosine (anti-PY) monoclonal antibody 4G10 (upper blot) or polyclonal anti-DDR2 antibodies (lower panel). The positions of molecular markers (in kilodaltons) are indicated. Collagen I was used at different concentrations as indicated (in μg/ml). The experiment was performed three times with very similar results.

Abdulhussein et al. (2004) examined a number of DDR1 point mutants for receptor activation by collagen I. They found that mutation of Arg105 or Ser175 to alanine abolished DDR1 activation, in agreement with our structure. Intriguingly, however, mutation of Trp53 (corresponding to Trp52 in DDR2) did not have an effect on DDR1 activation, and deletion of several residues in loop L1 was required to abolish DDR1 activation by collagen I (Abdulhussein et al., 2004). These findings are difficult to reconcile with the critical role of Trp52 in collagen recognition by DDR2 (Figure 2C). To support our interpretation of the structure, we tested DDR2 W52A mutant constructs for collagen I binding and receptor activation. A soluble ectodomain construct with the W52A mutation was secreted at similar levels as the corresponding wild-type protein, but failed to bind to collagen in an established solid-phase assay (Figure 6A). Likewise, full-length DDR2 W52A expressed in 293 cells could not be activated by collagen (Figure 6B). We conclude that Trp52 is indispensable for collagen recognition and signaling by DDR2. Given that the GVMGFO motif is also the major binding site for DDR1 (authors' unpublished data), we find it difficult to believe that the corresponding tryptophan in DDR1, Trp53, is not required for receptor activation.

### Convergent Evolution of GVMGFO-Binding Sites

**Figure 7:**
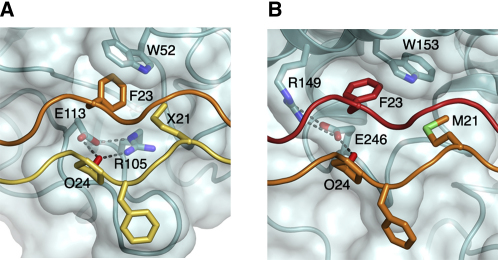
Comparison of Collagen Recognition by DDR2 and SPARC DDR2 (A) (this work) and SPARC (B) (Hohenester et al., 2008) are in cyan and shown as cartoons with semitransparent surfaces. The leading, middle, and trailing chains of the collagen peptides are in yellow, orange, and red, respectively. Selected residues are shown as sticks. X denotes norleucine. Dashed lines indicate hydrogen bonds.

The GVMGFO motif is a recently defined hotspot in collagens I–III that binds not only DDR2, but also von Willebrand factor (vWF) (Lisman et al., 2006) and the matricellular protein SPARC (Giudici et al., 2008). A comparison of the collagen complexes of DDR2 and SPARC (Hohenester et al., 2008) (a complex of vWF does not exist) reveals a remarkable case of convergent evolution. The GVMGFO-binding site of SPARC is created by a long α helix and an adjacent helical hairpin, in sharp contrast to the irregular loops that make up the binding site of DDR2 (Figure 7). Despite their different structures, however, both proteins feature similar amphiphilic specificity pockets, sandwiching the critical Phe23 side chain (middle chain in DDR2, trailing chain in SPARC) between a tryptophan and a salt bridge between arginine and glutamic acid, with the latter also mediating recognition of Hyp24. Another interesting parallel is that the Phe23 phenyl ring does not form any stacking interactions in either structure but is bound in a manner favoring the formation of C-H⋯π hydrogen bonds (Hohenester et al., 2008).

## Discussion

Cell-collagen interactions are critical for tissue stability and function, but structural studies are difficult because of the large size and structural complexity of collagens. Comprehensive sets of synthetic triple-helical peptides (“Collagen Toolkits”) have been invaluable in defining specific receptor-binding sites in collagens (Farndale et al., 2008) and have made possible crystallographic studies of receptor-collagen complexes. However, to date, α2 integrin has been the only collagen receptor for which the mode of collagen binding was understood in atomic detail (Emsley et al., 2000).

We have determined a high-resolution crystal structure of the DDR2 DS domain in complex with a 28-residue collagen peptide, revealing how DDR2 recognizes a conserved GVMGFO motif present in the fibrillar collagens I–III (note that in our peptide methionine is replaced by norleucine; see above). The two large apolar residues of this motif, M and F, are inserted into a specificity pocket at the top of the DDR2 DS domain. This pocket is surprisingly polar on one side, allowing multiple hydrogen-bonding interactions with the O of the GVMGFO motif. An important feature of the DDR2-collagen interaction, correctly predicted from modeling (Konitsiotis et al., 2008), is that the key collagen residues are not provided by the same chain, explaining why a triple-helical conformation is required for binding (Leitinger, 2003; Vogel et al., 1997).

Most remarkably, an essentially identical collagen-binding mode to DDR2 is employed by SPARC, an α-helical matricellular protein unrelated to DDR2 that also recognizes the GVMGFO motif in collagen (Giudici et al., 2008; Hohenester et al., 2008). The convergence of binding mechanisms suggests that the GVMGFO motif may have been selected as a binding site because of its unique properties: the presence of two large apolar residues separated by a glycine is rare in collagens and results in pronounced hydrophobic knobs on the triple helix surface.

Apart from the GVMGFO motif, which is present in Collagen II Toolkit peptides 22 and 23, additional DDR2-binding sites have been observed (but not yet characterized) in peptides 13 and 44 (Konitsiotis et al., 2008). A GIVGLO motif in peptide 44 may bind DDR2 in a similar way as the GVMGFO motif, but there are no analogous candidate motifs in peptide 13. Thus, alternative modes of collagen recognition by DDR2 may exist.

The major binding site in collagens I–III for α1β1 and α2β1 integrins is a GFOGER motif (Knight et al., 2000; Xu et al., 2000). In contrast to the situation with DDR2 and SPARC, all three phenylalanine side chains of the triple-helical GFOGER peptide remain substantially solvent-accessible in the complex with the integrin α2 I domain (Emsley et al., 2000), consistent with the finding that the requirement for phenylalanine is not strict (Kim et al., 2005; Raynal et al., 2006). The invariant residue of all integrin-binding sites in collagen is a glutamic acid, which coordinates the magnesium ion bound to the integrin I domain (Emsley et al., 2000). Thus, the two major classes of collagen receptors in animals, integrins and DDRs, have evolved to bind collagen by very different mechanisms despite their shared affinity for GFO triplets.

Is the GVMGFO motif also the major DDR2-binding site in collagen fibrils? In this regard, it is worth noting that DDR2 binding to fibrillar collagen has yet to be demonstrated by direct observation. However, fibrillar and nonfibrillar collagen have been shown to act differently on cells in a DDR2-dependent manner (Wall et al., 2005). A low-resolution structure of the collagen I microfibril has been reported recently (Orgel et al., 2006). Two alternative models of a collagen fibril have been generated from this structure (Herr and Farndale, 2009; Perumal et al., 2008), with the GVMGFO motif being surface-exposed only in the model of Herr and Farndale (2009). However, the binding mode observed in our DDR2 DS-collagen peptide structure is not compatible with the crystalline structure of Orgel et al. (2006). It is possible that DDR2 binds to the more disordered, fluid-like regions that are known to exist in collagen fibrils (Hulmes et al., 1995).

How does collagen binding to the DDR2 DS domain lead to receptor activation? Many RTKs are believed to be dimerized by their ligands, which brings the cytosolic kinase domains into close proximity and facilitates the autophosphorylation reaction that is the first step in RTK signaling (Schlessinger, 2000). Certain RTKs, such as the epidermal growth factor (EGF) receptor, appear to become activated by structural rearrangements within a preformed dimer (Jura et al., 2009). The DDRs are constitutive dimers at the cell surface (Abdulhussein et al., 2008; Mihai et al., 2009; Noordeen et al., 2006). Furthermore, collagen peptides containing the GVMGFO motif activate DDR2 with the same slow kinetics as native collagen, suggesting that receptor clustering is unlikely to be the main mechanism of DDR activation (Konitsiotis et al., 2008). We envisage an activation mechanism that involves collagen-induced changes within a DDR dimer. It should be noted that our discussion only refers to the first step of transmembrane signaling, not the slow process by which full DDR phosphorylation eventually is achieved (which minimally also involves Src kinase) (Ikeda et al., 2002; Yang et al., 2005).

**Figure 8:**
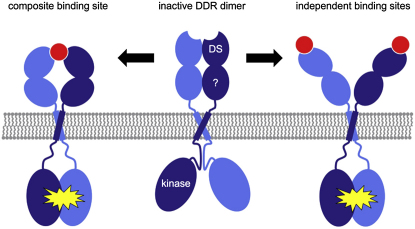
Possible mechanisms of DDR activation DDR1 and DDR2 are dimeric in the absence of collagen (Noordeen et al., 2006). The mechanism of autoinhibition in the inactive dimer is unknown, but is likely to involve the second domain of the ectodomain and/or the large cytosolic juxtamembrane domain. DDR activation may result from the simultaneous binding of both DS domains in the dimer to a single collagen triple helix (“composite binding site”), or the DS domains may bind collagen independently (“independent binding sites”). In any case, collagen binding is proposed to release the autoinhibition, resulting in activation of the cytoplasmic tyrosine kinase domains.

We can think of two ways in which collagen binding could activate DDR (Figure 8). A single collagen triple helix could interact with both DS domains in the DDR dimer (“composite binding site”) and thereby activate the receptor, similar to the situation exemplified by the growth hormone-growth hormone receptor complex (de Vos et al., 1992). The key collagen residues in our crystal structure are Nle21 and Hyp24 of the leading chain and Phe23 of the middle chain. Because of the helical symmetry of the homotrimeric collagen peptide, an equivalent constellation of residues occurs again on the middle and trailing chains (Figure 1B). However, it is impossible to replicate the interactions of the first DS domain at this second, vacant, site without causing major steric clashes between the two DS domains (data not shown). Thus, the complex would have to be asymmetric with two distinct receptor-ligand interfaces. The high-affinity DS-collagen interface would correspond to the interaction seen in our crystal structure, whereas the second interface may be weaker and only form when the two DS domains are joined in a stable DDR dimer.

In the alternative scenario, collagen binding to two independent sites would trigger the transition from the inactive to the active DDR dimer, conceivably by amplifying the small changes in the collagen-binding loops of the DS domain (Figure 3). This situation would be more akin to the EGF-EGF receptor complex, in which two EGF molecules bind to equivalent sites on the outside of an active receptor dimer (Burgess et al., 2003). It should be noted that there is no formal requirement for both DS domains to be occupied by ligand in the active DDR complex: binding of collagen to one DDR DS domain may be sufficient to “unlock” the inactive dimer. Structures of the full-length receptor will now be required to gain further insight into the mechanism of DDR activation. Our structure of a DDR2 DS-collagen complex provides the foundation for such future studies.

## Experimental Procedures

### Peptide Synthesis

Peptides were synthesized as C-terminal amides by the solid-phase method on a 9050 Plus PepSynthesizer (Perseptive Biosystems). The peptides were prepared on a 0.1 mmol scale, using Fmoc (9-fluorenylmethoycarbonyl) chemistry and TentaGel R RAM (Rapp Polymere) resin (0.18 mmol/g). Fmoc deprotection was performed using 2% (v/v) piperidine and 2% (v/v) 1,8-diazabicyclo-[5,4,0]undec-7-ene in dimethylformamide (DMF). Coupling of Fmoc-amino acids (0.4 mmol) was performed in DMF using HCTU (2-(6-Chloro-1-H-benzotriazole-1-yl)-1,1,3,3-tetramethyluronium hexafluorophosphate) (0.4 mmol) with *N*,*N*-diisopropylethylamine (0.8 mmol). Cleavage of the peptides from the resin and simultaneous side-chain deprotection was done by treatment of the peptide-resin with a trifluoroacetic acid (TFA), water, and triisopropylsilane mixture (95:2.5:2.5 v/v, 10 ml) containing DL-dithiothreitol (0.25 g), for 3 hr. The resin was filtered, and the filtrate was concentrated under reduced pressure to ∼1 ml volume, after which the crude peptides were precipitated with ice-cold ether. The filtered crude peptides were ether-washed (twice), dissolved in 5% acetonitrile in water containing 0.1% TFA, and then lyophilized. Crude peptides were purified by reverse-phase high-performance liquid chromatography (PerkinElmer Life Sciences LC200) using ACE diphenyl columns (Hichrom Ltd) and a linear gradient of 5–45% acetonitrile in water containing 0.1% TFA. The pure peptides were characterized by matrix-assisted laser desorption/ionisation-time of flight (MALDI-TOF) mass spectrometry and then lyophilized.

Acetylation of the peptide GPOGPOGPOGPRGQOGVNleGFOGPOGPOG-NH_2_ (Nle, norleucine) was performed manually. Following solid-phase synthesis and removal of the final *N*^α^-Fmoc protecting group, the prewashed resin was treated for 45 min with acetic anhydride (10 ml) and *N*,*N*-diisopropylethylamine (3 ml) in dichloromethane (10 ml). The filtered peptide resin was then washed with ether (four times). Cleavage from the resin, simultaneous side chain deprotection, isolation, and purification were performed as for the other peptides.

### Protein Expression Vectors

DNA coding for the DS domain of human DDR2 was obtained by PCR using the His-DDR2 vector (Leitinger, 2003) as a template. The DS domain boundaries used were NPAICR…CVWLDG, corresponding to residues 26–190 of SwissProt entry Q16832. The PCR product was cloned into a modified pCEP-Pu vector (Kohfeldt et al., 1997), which adds a His-tag (APLVHHHHHHALA) at the N terminus. The expression vector for the Fc-tagged DS domain (DS2-Fc) has been described elsewhere (Leitinger, 2003). The W52A mutation in full-length DDR2 was introduced by strand overlap extension PCR as described elsewhere (Leitinger, 2003); the PCR product containing the mutation was inserted into pcDDR2 using EcoRI and NarI. To create wild-type and W52A ectodomain constructs tagged with human Fc, cDNAs encoding the respective DDR2 ectodomains (Lys22-Thr398) were obtained by PCR amplification and cloned into a modified pCEP-Pu vector containing a human Fc sequence (Hussain et al., 2006). All expression vectors were verified by DNA sequencing.

### Protein Expression and Purification

All soluble DDR2 constructs were purified from the conditioned serum-free medium of episomally transfected 293-EBNA cells. Cells were cultured in Dulbecco's modified Eagle medium containing 10% fetal calf serum, (Invitrogen), transfected using Fugene reagent (Roche Applied Science), and selected with 1 μg/ml of puromycin (Sigma). Proteins were purified by a combination of affinity and size exclusion chromatography on an Äkta platform (GE Healthcare). The Fc-tagged proteins were purified using 1 ml rProtein A FF HiTrap columns according to the manufacturer's instructions (GE Healthcare) and were dialyzed into phosphate-buffered saline (PBS) buffer (140 mM NaCl, 10 mM Na_2_PO_4_, and 3 mM KCl [pH 7.45]). The conditioned medium containing the His-tagged DS domain was loaded onto a 5 ml HisTrap column (GE Healthcare) was equilibrated in PBS, and the DS domain was eluted with 500 mM imidazole in PBS. The eluate was concentrated using a Vivaspin centrifugal device (Sartorius AG), and the DS domain was further purified by size exclusion chromatography on a 24 ml Superdex 75 size-exclusion chromatography column (GE Healthcare) with 20 mM MES and 100 mM NaCl (pH 6.5) as the running buffer. The final yield from one liter of cell culture medium was 5 mg of DDR2 DS domain. The protein was only moderately soluble in PBS and a number of other buffers tested (≤1 mg/ml).

### Collagen Binding and Activation Assays

The solid-phase assay with immobilized collagen (peptides) and the DDR2 activation assay were performed as described elsewhere (Konitsiotis et al., 2008; Leitinger, 2003). For analytical size exclusion chromatography (column and running buffer as above), 16 nmol of purified DDR2 DS domain in PBS was mixed with varying amounts of collagen peptide, diluted from a concentrated stock solution, and incubated for 30 min in a total volume of 0.5 ml.

### Complex Formation and Crystallization

The DDR2 DS-collagen complex for crystallization was formed by dissolving the lyophilized peptide Ac-GPOGPOGPOGPRGQOGVNleGFOGPOGPOG-NH_2_ in 6 ml of a diluted protein solution (0.5 mg/ml protein; molar peptide:protein ratio ∼1.5:1). After incubation for 30 min, the solution was concentrated to a volume of 0.5 ml and subjected to size exclusion chromatography (column and running buffer as above). The DDR2 DS-collagen complex eluted as a single peak and was concentrated to 10 mg/ml. Crystals were obtained by hanging drop vapor diffusion at room temperature using 0.1 M PCB (pH 7.0) and 25% PEG1500 as precipitant. The PCB buffer system was produced by mixing sodium propionate (40 mM), sodium cacodylate (20 mM), and bis-Tris propane (40 mM) in a molar ratio of 2:1:2. Crystals grew as clusters that could be dissected into single crystals.

### Data Collection and Structure Determination

Crystals were flash-frozen in liquid nitrogen after a brief soak in mother liquor supplemented with 25% glycerol. Diffraction data were collected at 100 K on station I-02 at the Diamond Light Source (Oxfordshire, UK) at a wavelength of 0.980 Å, and processed with MOSFLM (www.mrc-lmb.cam.ac.uk/harry/mosflm) and programs of the CCP4 suite (CCP4, 1994). The DDR2 DS-collagen structure was solved by molecular replacement with PHASER (McCoy et al., 2005; Storoni et al., 2004) using the first DS domain (b1 domain) of neuropilin-2 (pdb 2qqj) (Appleton et al., 2007) and a truncated collagen peptide from the SPARC-collagen complex (pdb 2v53) (Hohenester et al., 2008) as search models. Completion of the model was aided by the solution structure of the free DDR2 DS domain (pdb 2z4f) (Ichikawa et al., 2007). Multiple rounds of rebuilding with O (Jones et al., 1991) and refinement with CNS (Brunger et al., 1998) resulted in a R-factor of 0.204 (*R*_free_ 0.230). The final model comprises DDR2 residues 27 to 187, all collagen residues except for Gly31 of the trailing chain, and 166 water molecules. Analysis with MOLPROBITY (Davis et al., 2004) shows that 98% of residues are in favored regions of the Ramachandran plot and that there are no outliers. Crystallographic statistics are summarized in Table 1. The figures were made with PyMOL (www.pymol.org).

## Figures and Tables

**Table 1:** Crystallographic Statistics of the DDR2 DS-Collagen Complex

Data collection	
Space group	P1
Unit cell dimensions	
a, b, c (Å)	33.40, 40.86, 48.92
α, β, γ (°)	66.19, 88.90, 76.92
Complexes/asymmetric unit	1
Solvent content (%)	42
Resolution (Å)	20−1.6 (1.69−1.60)^a^
R_merge_	0.046 (0.281)
<I/σ(I)>	16.8 (4.3)
Completeness (%)	90.2 (67.4)
Multiplicity	3.8 (3.8)
*Refinement*	
Resolution (Å)	20−1.6
Reflections	27359
Protein atoms	1293 (DS domain) + 552 (collagen)
Solvent atoms	166
R_work_/R_free_	0.204/0.230
Rmsd bonds (Å)	0.005
Rmsd angles (°)	1.5

a Values in parentheses are for the highest resolution shell.

## Accession Numbers

Atomic coordinates and structure factors have been deposited in the Protein Data Bank under the ID code 2wuh.
